# Evolution of γ chain cytokines: Mechanisms, methods and applications^☆^

**DOI:** 10.1016/j.csbj.2022.08.050

**Published:** 2022-08-25

**Authors:** Magdalena Antczak, Pablo F. Cañete, Zhian Chen, Clémence Belle, Di Yu

**Affiliations:** a The University of Queensland Diamantina Institute, Faculty of Medicine, The University of Queensland, Brisbane, Queensland, Australia; b Ian Frazer Centre for Children’s Immunotherapy Research, Child Health Research Centre, Faculty of Medicine, The University of Queensland, Brisbane, Australia

**Keywords:** γ chain cytokines, Interleukins, Immune system, T cells, Immunotherapies, Molecular evolution, Whole genome duplication, Tandem duplication, Pathogen-host co-evolution, Phylogenetic trees, Protein topology, Amino acid conservation, Positive selection, Multiple sequence alignments

## Abstract

The common γ chain family of cytokines and their receptors play fundamental roles in the immune system. Evolutionary studies of γ chain cytokines have elegantly illustrated how the immune system adapts to ever-changing environmental conditions. Indeed, these studies have revealed the uniqueness of cytokine evolution, which exhibits strong positive selection pressure needed to adapt to rapidly evolving threats whilst still conserving their receptor binding capabilities. In this review, we summarise the evolutionary mechanisms that gave rise to the characteristically diverse family of γ chain cytokines. We also speculate on the benefits of studying cytokine evolution, which may provide alternative ways to design novel cytokine therapeutic strategies. Additionally, we discuss current evolutionary models that elucidate the emergence of distinct cytokines (IL-4 and IL-13) and cytokine receptors (IL-2Rα and IL-15Rα). Finally, we address and reflect on the difficulties associated with evolutionary studies of rapidly evolving genes and describe a variety of computational methods that have revealed numerous aspects of cytokine evolution.

## 1 Introduction

In 1973, evolutionary biologist Theodosius Dobzhansky published a seminal essay titled “*Nothing in biology makes sense except in the light of evolution*” [1]. While the purpose of his words may have been to refute those arguing against evolution, this statement undeniably reconciles the many unifying biochemical features shared by all forms of life [2]. Since then, the field of molecular evolution has strived to understand the evolutionary trajectories of genes and proteins of interest. While highly conserved genes may reveal molecular pathways that, even if slightly altered, are simply unable to sustain life, divergent proteins often illustrate dynamic systems that require continuous adaptation to environmental triggers, such as the immune system [3].

### 1.1 Why study the evolution of the γ chain cytokines?

The immune system is an intricate network of organs, cells and molecules dedicated to protecting the host from foreign invaders and maintaining homeostasis. Given the complex and diverse entities that comprise it, this system must rely on effective communication mechanisms. Consequently, the body has evolved a large arsenal of soluble factors that mediate immune crosstalk and allow the immune system to fine-tune its functions [4]. These mediators, known as cytokines, utilise matching cytokine receptors that initiate downstream signalling cascades in target cells. Subsequent activation of signalling pathways ultimately promotes the expression of genes required for specific cellular processes. Cytokines perform diverse roles and virtually control most physiological processes, from embryonic development to haematopoiesis [5], [6], [7], [8], [9], [10]. This is possible due to their pleiotropic nature (i.e. ability for single cytokines to carry out multiple biological processes) as well as redundancy (i.e. the ability of multiple cytokines to exert similar actions). Therefore, tight control mechanisms that maintain cytokine balance are needed to fine-tune immune coordination and homeostasis. Indeed, dysregulation of cytokine signalling results in a wide range of pathological conditions, including primary immunodeficiencies, allergies, autoimmunity, cancers and cytokine storms characteristic of certain infectious diseases (e.g. SARS-CoV-2) [11], [12], [13], [14]. Interestingly, cytokine receptor deficiencies seem more detrimental than individual cytokine defects [15], [16], [17], [18], [19]. This is not surprising given the relatively large number of cytokines that share common cytokine receptors. Therefore, cytokines and their receptors are important determinants of health and disease and thus represent attractive therapeutic targets for a wide range of diseases [20].

Here we summarise and integrate investigations related to the evolutionary origins of the common γ chain family of cytokines. We highlight cytokines and receptors that have taken the spotlight in the scientific community, such as IL-2, IL-15, IL-2Rα, IL-15Rα, IL-21, IL-4 and IL-13. We illustrate various mechanisms that have shaped the manner and the extent to which these cytokines have evolved (e.g. host-pathogen arms races, whole genome duplication events). Furthermore, we present popular *in silico* tools used to interrogate molecular evolution hypotheses and assess cytokine evolutionary trajectories. Lastly, we speculate that gathering evolutionary insight into a family of proteins present in virtually all organisms whose immune system relies on adaptive immunity may reveal novel approaches to design cytokine-based immunotherapies.

### 1.2 Cytokine homologues as potential therapeutic agents

Cytokine administration is arguably the therapeutic approach that pioneered the field of immunotherapy. Seminal studies reported that treatments with TNF-α and IL-2 provided favourable outcomes in a variety of cancer settings [21], [22], [23], [24]. Such discoveries have laid the foundation for tweaking the immune system towards a pro-inflammatory state with the hope of achieving tumour regression. While animal studies have generated compelling and promising data, clinical translation has been slow and hampered by, at least in part, the toxicity of these regimes [22], [23], [25]. Thus, efforts to modify cytokine structure have been proposed to help mitigate toxicity and potentiate beneficial outcomes [26], [27], [28], [29], [30]. Most synthetic cytokine approaches involve the use of additional macromolecules linked to the cytokine itself [31], [32], [33], [34], [35]. However, very few studies report on the use of synthesising mutated cytokine versions that might be superior in providing favourable therapeutic outcomes. Given that generating and screening randomly mutated cytokines may be labour intensive and costly, it is tempting to speculate that testing cytokine orthologues might be a useful approach to reveal the principles of receptor-ligand co-evolution, which will help design cytokine modifications with beneficial properties.

### 1.3 Cytokine classification

Cytokine classification methods by either function or structure have revealed a myriad of cytokine families, such as chemokines, interferons, lymphokines, tumour necrosis factors and interleukins [4], [36], [37]. Interleukins are a group of cytokines that are foremost modulators of immune and inflammatory responses [37], [38], [39], [40], [41], [42], [43]. Even though they were once thought to be secreted by white blood cells only, interleukins have later been shown to be produced by numerous cell types other than the hematopoietic lineage. To date, more than sixty cytokines have been classified as interleukins [44], and six of them (i.e. IL-2, IL-4, IL-7, IL-9, IL-15 and IL-21) comprise the common γ chain family of cytokines, named after the γ chain (γ_c_) receptor they all bind [20], [45], [46]. The common γ_c_ cytokine receptor is essential for innate and adaptive immunity [20]. Loss-of-function mutations in humans result in X-linked severe combined immunodeficiency (X-SCID), an immune disorder with dysfunctional B cells and near absent T cells and natural killer (NK) cells [19]. Mice deficient for γ_c_ recapitulate human X-SCID with underdeveloped thymus and diminished B, T, and NK cells [47]. Likewise, the common γ_c_ family of cytokines play pivotal roles throughout the lifetime of various immune cell lineages, such as IL-7, which is fundamental for T cell development and function [48]. Similarly, IL-4 is key in mediating anti-parasitic and allergic responses [49], [50], whilst IL-2 controls T cell proliferation [51], and IL-21 is fundamental for orchestrating humoral responses [52]. Therefore, common γ_c_ cytokines are essential in mediating unique and diverse facets of the adaptive immune system.

## 2 Evolution of interleukins

### 2.1 When did interleukins first appear?

Given the pivotal role of interleukins in lymphocyte biology, the origin of interleukins is often attributed to the emergence of adaptive immunity. Adaptive immunity is often considered to have originated in the common ancestor that preceded early jawed vertebrates [39], [53], [54]. This ancestor gave rise to two distinct clades, cartilaginous fish (i.e. sharks) and bony vertebrates. Within the latter clade, the teleost family of fish (a large group of fish representing 96 % of all current fish) is often referred to as the oldest living fish containing an adaptive immune system similar to that of mammals [55], [56]. Thus, comparative analyses using these organisms are commonly used to illustrate the evolutionary timeline of cytokines. Whilst the emergence of interleukins coincides with that of the adaptive immune system [39], [57], evidence indicates their presence in jawless vertebrates (IL-13Rα1, IL-17) and even invertebrates (IL-6, IL-17) [39], [57], [58], [59]. Interestingly, the common γ_c_ family of cytokines are only found in jawed vertebrates [39], [53], [60], [61], suggesting they may have originated and evolved hand in hand with adaptive immunity.

### 2.2 Why do we have so many different cytokines?

#### 2.2.1 Several rounds of whole genome duplication

It is now widely accepted that the vertebrates’ common ancestor went through two rounds of whole genome duplication (WGD) [62], [63]. In humans, the majority of genes encoding short-chain type I cytokines (family containing γ_c_ cytokines) are located on chromosome arms 4q and 5q, which are paralogues dating back to the bifurcation of fish and tetrapods [63], [64], [65], [66], [67]. Consequently, a release of selective pressure rendered duplicated genes prone to accumulate mutations, which eventually led to divergent sequences and functions [53], [68], [69]. In addition, teleosts experienced a third round of WGD and salmonids (a family of teleosts) – a fourth one [56], [62], [70], [71]. These two fish-specific WGD rounds resulted in multiple paralogues of several common γ_c_ cytokines, such as IL-2 or IL-4/13 [64], [72], [73].

#### 2.2.2 Host-pathogen interactions

Cytokines (including the common γ_c_ family of cytokines) are among the fastest evolving genes. Indeed, seven out of the 25 fastest evolving genes with the highest degree of evolutionary divergence in mouse *vs* human orthologues code for cytokines or their receptors [54]. Such rapid evolution may be explained by gene duplication events and host-pathogen co-evolution. This is not surprising given the breakneck speed at which pathogens evolve and the relatively shorter generation times that allow them to rapidly adapt to the host. Additional clever adaptation strategies employed by pathogens include molecular mimicry, allowing the invader to remain unnoticed and to evade immune defence mechanisms [74], [75], [76]. Therefore, the host’s immune genes must evolve to counteract these adaptation strategies [74], [75], [76]. Several instances of such co-evolution have been reported. For example, some immunodeficiency viruses can copy several exact sites of IL-2 into the transmembrane envelope of their glycoproteins [76], [77], which confers them an ability to redirect antibody responses towards IL-2 instead. As a result, auto-IL-2 antibodies are typically detected in HIV patients. Additionally, recent COVID-19 studies have revealed that the SARS-CoV-2 open reading frame 8 (ORF8) glycoprotein resembles IL-17A [78]. Indeed, this viral protein has been demonstrated to bind the IL-17 receptor, which results in a more powerful inflammatory reaction than that induced by IL-17. Therefore, host-pathogen interactions have played a major role in painting the evolutionary canvas of many immune-related genes, including the γ_c_ family of cytokines. Gathering evolutionary insight of the latter may reveal novel sequences that can better modulate the immune response and thus may offer innovative and attractive therapeutic approaches.

## 3 Molecular evolution of γ_c_ cytokines and their receptors

Molecular evolution is the field of study that aims to delineate evolutionary trajectories of the biochemistry of life. A major theme in the field investigates whether mutations that confer evolutionary advantages sweep a population of interest [79], [80], [81]. Such inferences can be reached via studying the conservation of homologous gene sequences and interrogating whether a common ancestor sequence might be present. For example, in order to elucidate whether the common γ_c_ family of cytokines experience positive selection pressure, several studies compared the rates of non-synonymous *vs* synonymous substitutions in the sequences of γ_c_ cytokines across species [39], [62], [74], [75], [82], [83], [84], [85], [86], [87]. Whereas a higher incidence of non-synonymous mutations indicates adaptational positive selection pressures, a higher rate of synonymous mutations is indicative of the opposite. Not surprisingly, the abundance of non-synonymous variants in many sequences of γ_c_ cytokines across species suggest that this group of cytokines have evolved under positive selection pressure [82], [83].

### 3.1 Sites of positive selection

Identification of positive selection sites, which are likely correlated with sites of significant biological activity, can effectively determine the acquisition of mutations associated with competitive fitness [76], [84]. Several lines of evidence have identified positive selection sites in all six γ_c_ cytokines [82], [84]. The majority were found to be at or near the receptor-binding domains, suggesting that such sites may have granted these cytokines a competitive advantage in recruiting their receptor chains [82]. Indeed, studies that have identified extensive positive selection sites in *IL4*, which is paramount in mediating immunity to extracellular pathogens, illustrate the need for this pathway to keep up with recurrent exposure to parasitic worms [75], [85]. Nevertheless, conflicting evidence that contradicts this notion was generated by Kubick et al. in 2021, who suggested that both the IL-2 family (encompassing IL-2, IL-7, IL-9, IL-15 and IL-21) and IL-4 family (comprising IL-4 and IL-13) evolved under negative selective pressure [39]. In contrast to the high variability and genetic diversity observed across all γ_c_ cytokines, evolution has selected against diversity in the γ_c_, which is at the core of all the common γ_c_ family of cytokines and their receptors. Indeed, an abundance of negative selection sites at this locus suggests that γ_c_ is under strong pressure to remain unchanged, and not surprisingly, loss of function mutations in humans lead to one of the most severe immunodeficiency syndromes [82].

Even though most γ_c_ cytokines contain positive selection sites [74], [83] an ancestral gene that shares properties with *IL2* and *IL15*, thus termed *IL15-like* (*IL15L*), appears to exhibit characteristics of negative selection in most mammals [61]. IL-15L was firstly identified in fish as an IL-15Rα-binding cytokine, and while its presence in mammals remained elusive for decades, a genomic locus corresponding to fish *IL15L* was later confirmed in several mammalian species, such as cattle, pigs and horses [61]. This suggests that not only might *IL15L* mediate important functions in those species but also that conservation of this gene might have played beneficial roles throughout natural selection. It is interesting, however, that only remnants of this gene have been found in rodents and higher primates, and given its impaired open reading frame, a putative function for *IL15L* is highly unlikely in these species [61], [88], [89], [90]. Given the stark difference in evolutionary outcomes of *IL15L* in a single class of closely related vertebrates, it is tempting to speculate that pathogen and disease tropism may have been key drivers for such opposing evolutionary trajectories.

### 3.2 Challenges in comparative cytokine studies

Cytokine genetic divergence resulting from i) the many duplication events and ii) rapid accumulation of mutations due to host-parasite co-evolution makes comparative cytokine (and their receptors) studies troublesome. This is further hampered by the difficulty of designing PCR primers to isolate putative cytokines in new species. For example, it took seven years to identify IL-2 in chickens due to having only one reference sequence derived from mammals [91]. Efforts to isolate cytokines in marsupials were also initially unsuccessful and resulted in a long-standing notion that depicted the marsupial immune system to be rather primitive [91]. Furthermore, the lack of high sequence similarity across cytokine homologues and orthologues hinders bioinformatic algorithms that automate genome annotation. For example, Ensembl’s annotation pipeline (primarily using similarity of protein/RNA/DNA sequence and search/alignment tools that allow detection of only close homologues) has been able to detect only a handful of cytokines in marsupial genomes [91], [92]. Thus, alternative study methodologies and experimental designs are needed to evaluate the evolutionary history of γ_c_ cytokines.

### 3.3 Methods for studying molecular evolution

A variety of bioinformatic tools have been employed to draw parallels between the conservation of the common γ_c_ family of cytokines and their evolutionary trajectories across species. These exercises rely on the overall premise that proteins exhibiting similar sequences evolved from a common ancestor. Comparative multiple sequence alignments (MSAs), which essentially measure amino acid sequence conservation, have effectively revealed close/distant homologues and common protein ancestors. For example, conservation of cysteine residues and WSXWS motifs were used to classify some proteins as class I cytokine receptors [62], [93], [94]. Additionally, much of our understanding of the molecular evolution of cytokines has been aided by protein topology assessment (domains and motifs) as well as phylogenetic analyses [39], [53], [64], [73], [75], [76], [60], [61], [62], [93], [94], [95], [96], [97], [98], [99], [100], [101], [102], [103]. Indeed, phylogenetic trees constructed from mammalian IL-2Rαs and IL-15Rαs and fish IL-2/15Rαs allowed for clustering of close homologues and shed more light on which of the two mammalian sushi receptors originated from the primordial IL-2/15Rα [60], [104].

Phylogenetic relationships within the common γ_c_ family of cytokines are typically constructed by either rapid clustering methods, such as a neighbour-joining (NJ) algorithm, or by elaborate statistical algorithms like the maximum likelihood (ML) method [105], [106]. ML technique assumes an underlying substitution model of evolution, evaluates the probability of this model driving the evolution of the proteins in question and generally allows for detection of a more robust and accurate phylogeny. In addition, the reliability of a phylogenetic tree is often estimated via bootstrapping – a method that resamples and rebuilds a tree repeatedly [107]. The confidence value of a branch is calculated based on how many times the exact branch was reconstructed throughout the bootstrapping process.

Furthermore, next-generation sequencing methodologies, becoming increasingly affordable and practicable, have opened new research avenues to evolutionary molecular biologists. Identifying protein homologues across highly conserved sequences is relatively uncomplicated, and simple methods such as BLAST can typically produce meaningful data [108], [109]. However, for identification of homologues amongst more divergent sequences, tools that employ position-specific scoring matrices (PSSMs) or hidden Markov models (HMMs) may be more suitable [110], [111], [112]. These algorithms are built from multiple sequences using a specific family and incorporate the probability of amino acids being present at different positions for that family. In contrast, BLOSUM matrices, which are used by BLAST when comparing sequences across species, are based on overall amino acid frequencies and substitution probability [113]. Consequently, they allow less flexibility when searching for homologues than HMMs and PSSMs. In addition, gene synteny, which describes the physical co-localisation of genetic loci within a chromosome and across species, has revealed further conservation of the γ_c_ cytokines across several vertebrates [60], [73]. Integrating HMMs with gene synteny has been a fruitful approach, as many cytokines in the opossum genome initially missed by Ensembl’s automated annotation pipeline have been elucidated through this methodology [91], [114]. Moreover, gene synteny has significantly improved identification of IL-2 and IL-15 in many tetrapod and teleost species of fish [61], [64], [76], [100], [115], [88], [89], [90].

Finally, an alternative approach that may reveal molecular evolutionary insight utilises protein tertiary structure. It has been shown that some γ_c_ cytokines fold into similar structures, and despite abundant genetic sequence dissimilarities, their tertiary structure appears to be conserved throughout evolution [116]. However, although structural analysis is an attractive tool to reveal molecular evolutionary insight, its use has remained relatively scarce. This is partly due to the lack of experimentally-validated protein structures of the common γ_c_ family in many species but also the impracticality of elucidating all crystal structures for all the known γ_c_ receptors. Furthermore, until 2018, computational methods predicting protein’s structure were not highly conclusive or reliable [117], [118]. Nonetheless, the advent of novel algorithms that can predict tertiary and quaternary protein structures reliably, together with emerging machine learning tools, will certainly pave the way for a new era of molecular evolution.

### 3.4 Comparative evolution studies

#### 3.4.1 IL-7

IL-7 is arguably-one of the most important common γ_c_ chain cytokines in mammals and higher vertebrates. IL-7-deficient mice exhibit a 20-fold decrease in T cell numbers, and abrogating the IL-7 receptor (IL-7Rα) leads to virtually no T cells and B cells [119], [120], [121]. In contrast, both IL-7 and IL-7Rα deficiencies in humans result in severe T cell lymphopenia while retaining normal B cell numbers [122]. Despite the central roles of IL-7 in mammalian T cell development and function, IL-7 appears redundant in more distant vertebrates [48], [123]. Indeed, IL-7-deficient zebrafish only display a moderate decrease in thymocytes [119], [120], [123], [124], suggesting an evolutionary trajectory from degenerate to non-redundant roles of IL-7 in T cell development and function in higher vertebrates [123]. Nevertheless, the IL-7 signalling axis has remained fundamental throughout evolution, and despite the differing roles of IL-7 across species, IL-7Rα deficiency leads to equally catastrophic consequences in most organisms [48], [121], [122].

#### 3.4.2 IL-2, IL-15 & IL-21

Homologues of human IL-2, IL-15 and IL-21 have been successfully identified across mammals, birds, reptiles, amphibians and fish (both cartilaginous and bony fish species) [60], [61], [72], [76], [83], [91], [99], [100], [114], [125], [126], [127]. In addition, elegant studies have revealed the existence of another IL-15Rα-binding cytokine, IL-15L. While originally identified in fish, this cytokine is also present in several mammalian species. Despite the lack of evidence for an immunological function of IL-15L in mice and humans [61], [88], [89], [90], it may have contributed to the evolution of mammalian IL-2 and IL-15 [61].

IL-2, IL-15 and IL-21 exhibit a high degree of homology [125], and they all share a sequence motif absent in other short-chain helical cytokines [61]. Notwithstanding, some residues that are well conserved throughout IL-2, IL-15 and IL-15L seem absent in IL-21. Such residues mediate IL-15:IL-15Rα binding, thus providing a plausible explanation as to why IL-21 does not bind a sushi domain-containing receptor [61], [128]. Furthermore, human and mouse phylogenetic trees depicting the common γ_c_ family of cytokines identified close relationships between IL-15 and IL-2, which in turn share the closest common ancestor with IL-21 [129], [125]. Similar approaches have revealed distinct clusters for each of these cytokines [60], with the exception of teleost fish IL-2 proteins. The latter appears to be in closer proximity to other teleost fish IL-15 proteins than to mammalian IL-2 sequences. This phenomenon is also present in grass carp IL-2, which, compared to human γ_c_ cytokines, is revealed to be closer to human IL-15 than with IL-2 [60]. It is worth noting that others have produced contradicting results, proposing that carp IL-2 and IL-15 sequences are clustered with mammalian IL-2s, whereas IL-15 orthologues are more similar to IL-21 than to IL-2 [72].

Genomic co-localisation of *IL2* and *IL21* is well conserved across all vertebrates. They are tandemly clustered in fish, amphibians, reptiles, birds and mammals [60], [61], [72], [76], suggesting that they likely originated from a duplicated ancestor gene [76], [130], [131]. However, duplicated genes are not necessarily maintained in close proximity throughout evolution. For instance, *IL15* and *IL15L* do not physically co-localise, even if *IL15* sits on the same chromosome as *IL2* and *IL21* in many species (for example, humans, cattle, opossum or gar) [61], [125]. The fact that *IL2* and *IL21* are adjacent in species that bifurcated 500 million years ago poses an interesting yet puzzling question. Whilst conservation of this genomic arrangement may suggest an evolutionary advantage, it is difficult to envisage one given that these cytokines 1) exert diverse and often opposing functions, 2) co-expression is uncommon, and 3) they are differentially regulated [132], [133], [134].

Much debate regarding the origins of these three cytokines has resulted in several plausible scenarios. Bird et al. have suggested that there may have been an *IL2*/*IL15*/*IL21* primordial gene which, upon some duplication event with subsequent gene speciation mechanisms, gave rise to the three distinct cytokines [76]. Alternatively, Dijkstra et al. have proposed alternative origins for the *IL2*/*IL15* ancestral gene based on the conservation of cysteine residues. All vertebrates appear to harbour four key cysteine residues in IL-15. In contrast, IL-2 has four cysteine residues only in pufferfish and chicken, while mammalian IL-2s possess only two conserved cysteine positions. This led the authors to speculate that the precursor for IL-2 and IL-15 may have duplicated even before bony fish evolution [61]. Additionally, co-localisation of *IL2* and *IL21* across vertebrates from bony and cartilaginous fish to humans advocates for an *IL2* and *IL21* precursor that was also duplicated in early-jawed vertebrates.

#### 3.4.3 IL-2Rα *vs* IL-15Rα

Genomic co-localisation of *IL2RA* and *IL15RA* can give us clues about their evolutionary history. These two genes are tandemly clustered in a syntenic region containing *ANKRD16*, *FBH1*, *IL2RA*, *IL15RA* and *RBM17* in humans and birds [60]. This arrangement is also conserved in various tetrapod genomes such as mice or African clawed frogs [135], [60], [61], [62]. However, only a single copy of this receptor is found in the corresponding locus of fish. This suggests three possible scenarios for a putative common ancestor: IL-2Rα, IL-15Rα or a protein with a high degree of similarity to both, named IL-2/15Rα [72], [99], [60], [61], [62]. Elegant modelling has demonstrated the presence of IL-2Rα in the West Indian Ocean coelacanth (a lobe-finned fish) and IL-2/15Rα in several species of ray-finned fish and the Australian ghostshark (cartilaginous fish) [60]. Whether it is IL-2Rα or IL-15Rα that occupies the locus mentioned above varies and seems to depend on which receptor the research group attempts to identify. In 2007, IL-15Rα was cloned for the first time in a rainbow trout [104]. In 2011, attempts to isolate a homologue of IL-2Rα in a tetraodon (a teleost) resulted in the unprecedented discovery of a receptor that binds both IL-2 and IL-15 [136]. Further research on IL-15Rα and IL-2Rα identified homologues of IL-15Rα in gar (a ray-finned fish) and Australian ghostshark [61]. However, homologues for both receptors were ultimately identified in mammals, reptiles, amphibians, and fish – specifically ray-finned fish [39].

**Fig. 1:**
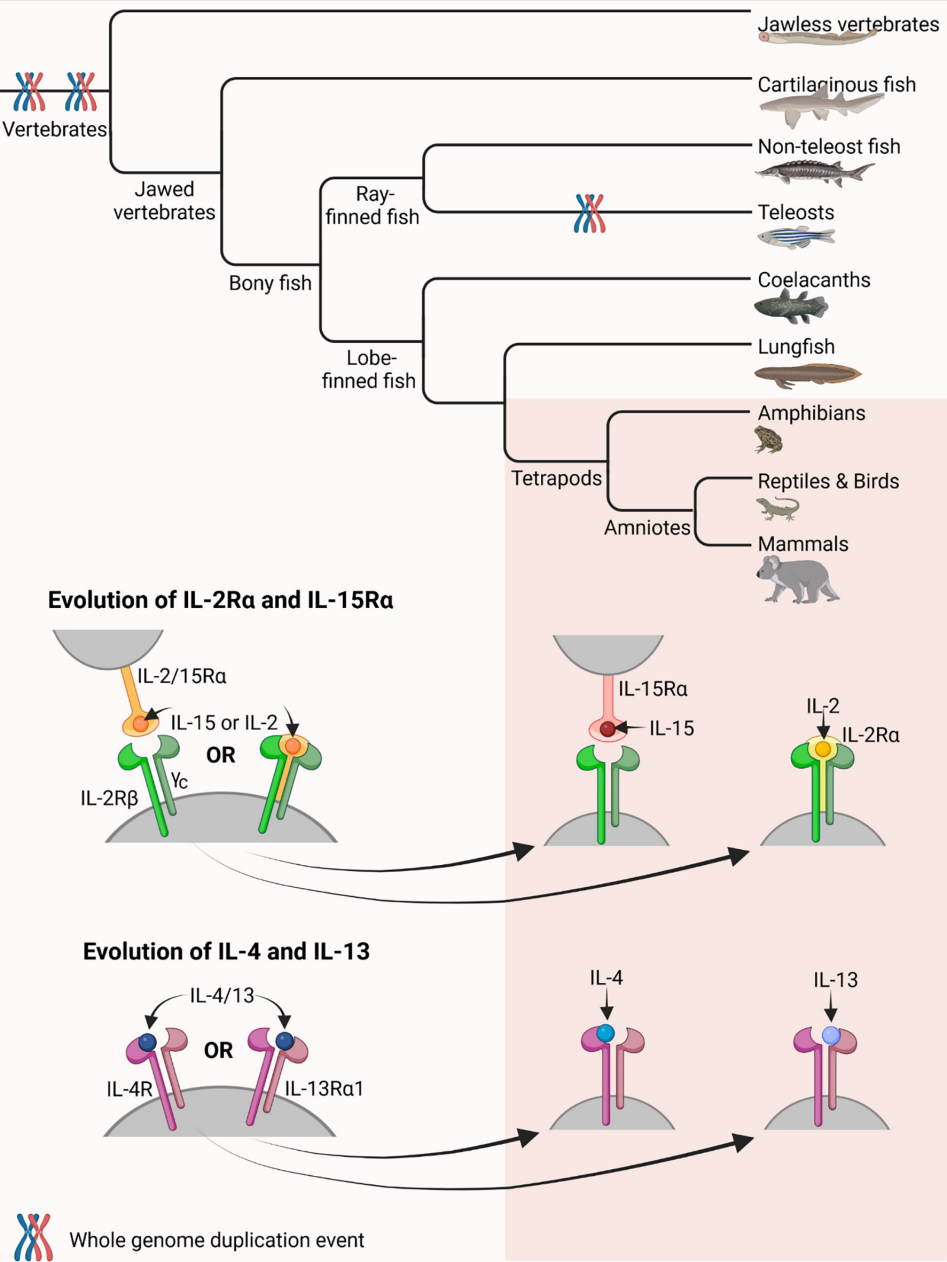
**Evolutionary models of IL-15Rα *vs* IL-2Rα and IL-4 *vs* IL-13.** Both models hypothesise that primordial genes coding for IL-2/15Rα and IL-4/13 underwent a tandem duplication event followed by acquiring overlapping yet distinct functions after fish and tetrapods separated. These models were presented by Wen et al., Wang et al. and Heeb et al., respectively [53], [60], [136]. The phylogeny of vertebrates shown here is adapted from a figure provided by Yamamoto et al. [137]. Darker background emphasises the presence of separated IL-15Rα, IL-2Rα, IL-4 and IL-13 in tetrapods only (according to the current evidence). Created with BioRender.com.

In summary, the evolutionary trajectory of IL-2Rα and IL-15Rα in tetrapods eludes to a model where they originated from the IL-2/15Rα receptor found in fish (Fig. 1) [60], [136]. Advocates for this model have formulated this hypothesis under the premise that a duplication event of IL-2/15Rα must have occurred in tetrapods after these two clades bifurcated. Consequently, the duplicated receptor was relieved from selective pressure and gained an additional sushi domain to facilitate binding to IL-2.

#### 3.4.4 IL-4 *vs* IL-13

Both IL-4 and IL-13 are primarily secreted by T helper 2 (Th2) cells, and in mammals, they play a major role in allergic reactions and immune responses against extracellular parasites [50], [73], [98]. They do so by promoting Th2 differentiation of CD4 + T cells, driving the production of high-affinity immunoglobulins of class E (IgE) and enhancing macrophage activation [49], [73], [98], [138], [139]. These cytokines also mediate repressive functions of other major immune responses, such as Th1 and Th17 effector functions. For instance, in mice infected with intracellular pathogens such as Leishmania major, IL-4 was shown to antagonise Th1-mediated inflammatory responses [138]. Similarly, in mouse models of Delayed-Type Hypersensitivity Reactions (DTHR) and human patients with psoriasis, systemic IL-4 administration curtailed differentiation and maintenance of Th17 cells [139]. Interestingly, lymphocytes are usually desensitised to IL-13 due to the low level of IL-13Rα1 expression [140], while *in vitro* polarised mouse Th17 cells exhibit elevated *Il13ra1* transcription compared to Th1 and Th2 cells [141]. IL-13 represses IL-17 and IL-21 production in *in vitro*-polarised Th17 cells, suggesting that IL-13 signalling suppresses Th17 responses [141]. The immunomodulatory effects of IL-4 and IL-13 also comprise repression of inflammatory innate immune cells, particularly neutrophils. For example, neutrophil infiltration in mouse skin upon cutaneous infection of bacteria is inhibited by administration of IL-4 and increased by IL-4-blocking agents [142].

While many roles of IL-4 and IL-13 overlap, they still exhibit differential functions [98]. Even though IL-4 and IL-13 are typically observed in many mammalian species as separate cytokines [64], [73], [98], [102], [125], only one homologue of IL-4/13 has been identified in many bony fish species (including teleosts and spotted gar) and cartilaginous fish (elephant shark). This may be due to the lack of IgE in fish. IgE mediates such potent inflammatory cascades [140], [141], [142] that perhaps a bifurcation of IL-4 and IL-13 was needed in order to serve as a tight regulatory layer in mammals. Interestingly, multiple copies of *il4/13* were found on different chromosomes in various teleost fish species, likely due to the additional WGD event in teleosts [73].

*IL4*, *IL13* and *IL4/13* are located in the Th2 locus control region, specifically in *KIF3A*/*IL4*/*IL13*/*RAD50* locus [73], [98]. This region is well conserved across many jawed vertebrates, including bony and cartilaginous fish. In humans and chickens, *IL4* and *IL13* lie side by side between the *KIF3A* and *RAD50* genes [73]. Similarly, *il4/13* (either single or multiple copies tandemly duplicated) lies in the well-conserved *kif3a*/*il4*/*il13*/*rad50* locus in frogs and two non-teleost fish species, spotted gar (bony) and elephant shark (cartilaginous) [98]. In addition, evolution seems to have conserved the genetic structure of all three Th2 cytokines discussed herein (*IL4*, *IL13* and *IL4/13*). They all harbour an intron/exon organisation typical for the short-helix type I cytokine family [64], [102], which suggests that this specific gene composition may be crucial for performing similar biological roles [143], [144].

The prevailing model of *IL4* and *IL13* evolution resembles the one suggested for an *IL2/15RA* primordial gene that bifurcated into *IL15RA* and *IL2RA* (Fig. 1) [53], [60], [64]. This model entails that the ancestral *IL4/13* gene present in early jawed vertebrates duplicated in tandem during vertebrate evolution and gave rise to distinct *IL4* and *IL13* loci [53], [64]. More studies in cartilaginous fish, bony non-teleost fish, amphibians and reptiles will have to be performed to identify when this duplication occurred.

## 4 Conclusions and future perspectives

Despite the lower cost and increasing affordability of genome sequencing, which together with gene synteny arguments have opened new avenues for studying molecular evolution, many questions regarding evolutionary trajectories of the common γ chain family of cytokines still remain elusive. Furthermore, the lack of characterisation of these cytokines in many species that originated from the vertebrate clade (apart from teleost fish and mammals) warrants further research to fill the gaps and enrich the evolutionary hypotheses presented in this review. In addition, most studies have only included a mere handful of organisms in their multiple sequence analyses and phylogenetic trees to draw conclusions, albeit the necessity to incorporate as many species as possible to unveil more reliable relationships between these cytokines and their receptors. We also postulate that the field would benefit from efforts that integrate state-of-the-art algorithms able to predict tertiary and quaternary protein structures when conducting comparative γ_c_ sequence analyses. This approach could potentially allow the scientific community to explore evolutionary mechanisms that simultaneously enable i) conservation of key interactions between cytokines and their receptors across vertebrates and ii) acquisition of changes needed to adapt to host-pathogen arms races. Finally, we propose the need to address nomenclature issues associated with newly identified proteins, which are particularly notable for rapidly evolving proteins with highly divergent sequences across organisms, such as γ_c_ cytokines. In such instances, we recommend discarding sequence similarity as a guideline to name novel proteins and consider a combination of protein topology and functional properties where possible.

## CRediT authorship contribution statement

**Magdalena Antczak:** Conceptualization, Investigation, Writing – original draft, Project administration. **Pablo F. Cañete:** Writing – review & editing. **Zhian Chen:** Writing – original draft. **Clémence Belle:** Visualization. **Di Yu:** Conceptualization, Resources, Supervision, Funding acquisition.

## Declaration of Competing Interest

The authors declare that they have no known competing financial interests or personal relationships that could have appeared to influence the work reported in this paper.
